# Liking as a balance between synchronization, complexity and novelty

**DOI:** 10.1038/s41598-022-06610-z

**Published:** 2022-02-24

**Authors:** Inbal Ravreby, Yoel Shilat, Yaara Yeshurun

**Affiliations:** 1 Department of Neurobiology, Weizmann Institute of Science, Rehovot, Israel; 2 The Azrieli National Institute for Human Brain Imaging and Research, Rehovot, Israel; 3 Department of Psychology, Ben-Gurion University of the Negev, Beer Sheva, Israel; 4 Zlotowski Center for Neuroscience, Ben-Gurion University of the Negev, Beer Sheva, Israel; 5 School of Psychological Sciences, Tel-Aviv University, Tel-Aviv, Israel; 6 Sagol School of Neuroscience, Tel-Aviv University, Tel-Aviv, Israel

**Keywords:** Human behaviour, Social behaviour

## Abstract

Synchronization has been identified as a key aspect in social bonding. While synchronization could be maximized by increasing the predictability of an interaction, such predictability is in tension with individuals’ level of interest, which is tied to the interaction’s complexity and novelty. In this study, we tested the interplay between synchronization and interest. We asked 104 female dyads to play the Mirror Game, in which they had to move their hands as coordinately as possible, and then report how much they liked each other. Utilizing information theory and video processing tools, we found that a combination of movement synchronization and complexity explained liking almost two times better than movement synchronization alone. Moreover, we found that people initiated novel and challenging interactions, even though they paid a price—being less synchronized. Examining the interactions’ dynamics, we found that people who liked each other moved in a more synchronized, complex, and novel manner during most of the interaction. This suggests that in addition to synchronization, maintaining interest may be critical for positive social bonding. Thus, we propose a new framework in which balancing synchronization and interest, rather than merely maximizing synchronization, optimizes the interaction quality.

## Introduction

Sensorimotor communication, which is achieved through modification of individuals’ body movements to communicate their intentions, is integral to human social communication and coordination^1,2^. It has been shown that during social interactions, dyadic coordination and synchronization of movements play a key role in successful interaction and achievement of mutual goals^3^. As sensorimotor communication is a multifaceted process, in this study we explored aspects beyond merely synchronization—movement complexity and novelty—that lead to positive social interactions in humans following a coordination task.

Synchronization of movements is defined as the similarity of movements^4^, either in the time domain or the frequency domain^5^. A large body of literature has shown a positive relationship between the quality of social interaction and synchronization of movements^5–8^. For example, when individuals interacted with others who mimicked their body language or when the participants are explicitly instructed to coordinate their movements, synchronization of movements was shown to increase liking^6,8,9^, affiliation^10^, rapport^11^, trust^5,12^ and collaboration^13^. Synchronization is so fundamental in humans’ interactions that even when people are instructed to ignore their partner, they get in sync anyway^14^. These results are related to a broader concept of synchronization that was suggested as a mechanism for social understanding^15,16^. Specifically, it was suggested that movement synchronization is a body-based way for creating socio-emotional connection and shared experiences^16,17^. In coordinated actions, or when mirroring each other, the actions of the self and the other overlap, and this facilitates mutual understanding^18^. Synchronization of body movements is also related to positive feelings such as connectedness and togetherness^10,17,19,20^. Remarkably, there is not only a correlation, but also a bidirectional causal relationship between social interaction quality and synchronization^19,21,22^. The aforementioned studies have established the role movement synchronization plays in successful social interactions. On the other hand, there is also evidence that reduced synchronization may result in better interaction, and specifically in secured attachment^23^ and even that the outcomes of movement synchronization on social interactions may be negative in a complicated joint task^24^, suggesting context dependency^24^. These findings point on the need for further research to better understand in a more nuanced manner the relationship between movement synchronization and positive social interaction. Here we suggest predictability as a main factor in this nuanced relationship.

Making oneself predictable may be used as a synchronization strategy. For example, when two acquaintances are approaching one another and want to greet each other, one person may intend a handshake, while the other may intend a hug. If one of the acquaintances starts raising the right hand when approaching the other, the partner would know in advance to match their movements so they will shake hands successfully. Indeed, research shows that predictable movements facilitate human synchronization during non-verbal interaction^25–27^. Thus, if individuals make predictable movements, their coordination increases since they can plan how to move properly, and coordinate their movements. Increasing predictability, therefore, may increase the likelihood of positive social interaction.

Notably, a predictable movement may be predictable in two distinguished aspects. One is the movement complexity in terms of what information may be known from time *t* on time *t* + 1. The other is the movement novelty in terms of how repetitive the movement is, i.e., what one may know from all previous movements on the next movement. Accordingly, these two dimensions of predictability—complexity and novelty—are independent in principle. A movement may be new but simple (not complex), or it may be complex but repetitive (not novel). Significantly, a highly simple or repetitive interaction is not necessarily rewarding, as the interaction might feel tedious^28,29^ and boring^30^. Boredom may be defined as the aversive experience of wanting a satisfying and challenging activity but being unable to engage in it^28,31^. Any dyadic interaction can be conceived as a dynamic process by which information is exchanged between individuals^17,32,33^. Following that, boredom may occur when there is a lack of rich information, making the activity unsatisfying. Such an experience is likely to occur during highly predictable interactions. One can avoid boredom by exploring novel stimuli—that is, a change in stimuli conditions from previous experience^34^. Moreover, individuals constantly strive for complexity and novelty^29,35^, as such patterns violate prior expectations, and thus may lead to more interesting and challenging interactions. This suggests that the more patterns are unpredictable (complex and novel), the more rewarding the interactions. This notion is consistent with Hasson and Frith^32^ who argued that we need to go beyond simple mirror alignment once we start interacting, and with Wohltjen and Wheatley^36^ who demonstrated the intricate dynamics of pupil dilation synchronization.

Altogether, we suggest a novel framework in which in order to optimize the interaction quality, there should be a subtle balance between being synchronized and generating interest. This implies that in contrast to the current view, maximizing the synchronization per se would not necessarily result in better interaction. Accordingly, in this study we set out to test the role of synchronization, complexity, and novelty in generating positive interactions, in the context of sensorimotor dyadic communication. We hypothesized that interesting movements, which are complex and novel, will improve the interaction more than mere synchronization. Moreover, we hypothesized that when people are asked to synchronize their movements with each other, they will choose to add complexity and novelty to the interaction, despite greater difficulty in arriving at synchronization.

## Results

**Figure 1:**
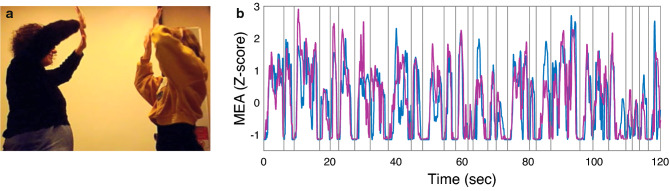
The Mirror Game. (**a**) A pair playing the Mirror Game while a hidden camera alarm clock, that was placed on a table next to the players, is filming the game. (**b**) The Z-scored MEA signals of two representative participants while playing with each other. The vertical lines denote the separation between the movement segments.

Participants were assigned to pairs and played the full-body Mirror Game^37^, in which they were only asked to freely move their hands with as much coordination as possible, without talking, for two minutes. There were no instructions regarding the direction of the movement or regarding the leader and the follower (see Fig. 1a). After each game, participants indicated how much they liked their partner (all liking ratings are available in the Liking data file). We filmed the games using a hidden camera and quantified each player's motions using motion energy analysis (MEA)^21,38^ (all dyads’ MEA results are available in the data files). Finally, the Z-scored MEA signal was analyzed in order to learn about the synchronization, complexity and novelty of the movements, and their relationship with liking (see “Methods” section for more details).

Next, to test whether dyads' movement dynamics during the interaction reflect liking, we divided the dyads according to the median into two groups^39,40^: the high-liking half and low-liking half, each comprising 50 dyads. We then compared synchronization, complexity, and novelty between the two groups along the time domain.

### Dyads' movement reflected reciprocal relationships

Granger causality analysis (GCA) is a method that uses autoregressive models to measure the causal relationship (i.e., Granger causality) between two time series^41^. This is done by testing whether one time series can predict the other above and beyond the auto-prediction of this time series, and vice versa^41^. We tested the pairwise-conditional GCA causalities of each participant's movement in the dyad. This procedure allowed us to examine whether within the dyads one member is the leader and the other is the follower or both members are interchangeably leaders and followers, so that each member’s movement influences the other’s movement. The GCA showed that in 95 out of the 100 dyads, each player's movement was significantly causally (Granger caused) determined by the other player's movement, i.e., there was a significant mutual Granger cause. These results suggest that the vast majority of the dyads, 95%, did not consistently play the role of leader or follower, but rather changed roles during the game^42^. In other words, in 95% of the dyads there was a bidirectional coupling. Such a symmetric and reciprocal relationship may be more rewarding^43^. Naively, one may expect the participants to spontaneously form a leader–follower pattern, believing the higher predictability will enhance their synchronization. However, only five out of the 100 dyads exhibited a unidirectional, leader–follower movement pattern, with no reciprocity. Perhaps surprisingly, this mutual adaptation was previously found to increase synchronization compared to leader–follower unidirectional adaptation, both in improvisation experts^20^ and non-experts^44^. Altogether, the results show that in all the dyads at least one participant Granger-caused the movements of the other, and, therefore, the partner mirrored their movements. This suggests that all dyads followed the instructions and moved in a coordinated manner, and that in the vast majority of them, there was bidirectional coupling and not unidirectional adaptation.

### Dyads chose to be novel and complex

**Figure 2:**
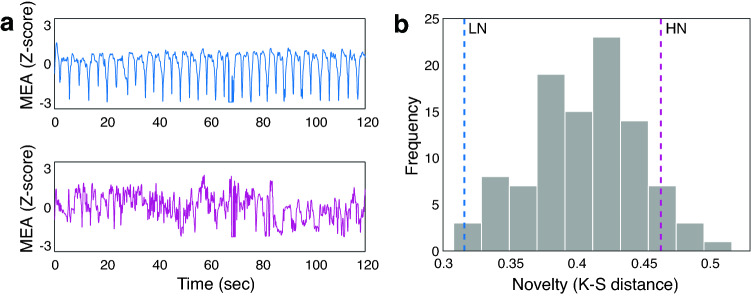
The dyads moved in a novel manner. (**a**) The upper panel shows the Z-scored MEA of repetitive up and down hand movements—low-novelty (LN) movement. The bottom panel shows the Z-scored MEA of diverse and novel hand movements—high-novelty (HN) movement. The hand movements were measured in a setup identical to that of the dyadic Mirror Games. (**b**) A histogram of the novelty of the dyads' movements during the Mirror Game. The blue dashed line denotes repetitive hand movements up and down (LN). The magenta dashed line denotes diverse and novel movements (HN).

Our results indicate that among all 100 dyads, there was not a single dyad in which participants repeated the same hand movements throughout the whole Mirror Game, according to a judge who watched the videos of the Mirror Game. To objectively test how novel (non-repetitive) the interactions were, we developed a new method for quantifying dyadic novelty. To do so, we first divided each interaction into movement segments by the stopping points (see “Methods” section for more details). We then calculated a dyadic novelty score comparing each segment to each of the previous segments using the Kolmogorov–Smirnov distance measure (K-S distance). Last, since the relative novelty differences within each dyad were very small (see Supplementary Material Fig. 4b), we averaged the novelty of all segments within each dyad (see “Methods” section for more details). To establish a reference point and get a sense of the dyads’ movement novelty, we compared it to an example of low- and high-novelty movements. This was done by filming a volunteer, who did not participate in the original experiment, twice: once while periodically moving their hands up and down for two minutes in a highly repetitive manner, and once while moving their hands in a highly innovative way, by varying their movement speed and angles, trying not to repeat any movement pattern, for two minutes. This was done using the exact same setup used in the Mirror Game experiment. We calculated the Z-scored MEA^21,24,45^ of these two videos and then calculated their novelty (using the K-S distance). The low-novelty (LN) movements are shown in the upper panel of Fig. 2a, demonstrating a highly repetitive signal, and the high-novelty (HN) movements are presented in the bottom panel of Fig. 2a, demonstrating a diverse and non-repetitive signal.

The novelty score we developed revealed that 99% of the dyads moved in a more novel way than just moving the hands up and down repeatedly (LN), even though we had not instructed them to perform novel movements. Furthermore, only 9% of the dyads moved in a more novel manner than when intentionally moving the hands in a highly diverse and novel manner (HN). This result further validates the novelty measure we chose, since our defined low-novelty measure (LN) was at the far-left side of the novelty histogram (0.32, as marked by the blue dashed line in Fig. 2b) and our definition for high-novelty movements (HN) was at the right side of the novelty histogram (0.46, as marked by the magenta dashed line in Fig. 2b). The mean novelty of all dyads = 0.4, *SD* = 0.04, and two-tailed one-sample t-test showed that low-novelty movements, repeated hand movements, were significantly lower than the average novelty *t*(99) = 21.84, *P* < 0.001, Cohen’s *d* = 2.18, 95% CI = [1.82, 2.54], BF_10_ > 1,000, whereas high-novelty movements, diverse hand movements, were significantly higher than the average novelty *t*(99) = 14.05, *P* < 0.001, Cohen’s *d* = 1.4, 95% CI = [1.13, 1.68], *BF*_10_ > 1,000. Thus, even though moving hands up and down repeatedly makes it easier to get into sync^26,27^ and is in accordance with the instructions, 99% of the dyads preferred moving in a less repetitive, more novel, and more interesting manner.

**Figure 3:**
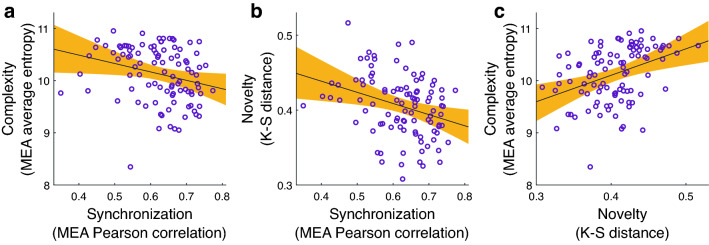
A tradeoff between synchronization and being interested. Pearson correlations between the different movement measures. In each panel each circle represents a dyad, and the black line is the linear regression line. The orange area marks the confidence interval around the slope of a regression line. (**a**) Negative correlation between complexity and synchronization, *r* = − 0.27, permuted *P* = 0.007, *P* = 0.003, 95% CI = [− 0.44, − 0.08], *BF*_10_ = 4.252. (**b**) Negative correlation between novelty and synchronization, *r* = − 0.32, *P* = 0.001, permuted *P* < 0.001, 95% CI = [− 0.48, − 0.13], *BF*_10_ = 21.88. (**c**) Positive correlation between complexity and novelty, *r* = 0.39, *P* < 0.001, permuted *P* < 0.001, 95% CI = [0.21, 0.54], *BF*_10_ = 344.21.

Next, we tested the relationships between the synchronization, complexity, and novelty of the dyads. Synchronization was measured using Pearson correlation (see “Methods” section for more details). Complexity was calculated using Shannon entropy^46^, which is defined as the average level of information. Information theory, the theoretical framework underpinning Shannon entropy, attributes higher levels of information to more unpredictable distributions. In other words, when an interaction is less predictable in terms of what one may know from time *t* on time *t* + 1, it is more complex^25,47^. Novelty was measured using K-S distance between each movement to the previous ones, enabling to assess the repetitivity of the movements (see “Methods” section for more details). To account for the fact that each participant was a member of more than one dyad, we performed permutation tests (see “Methods” section for more details). Accordingly, we report both the original *P* value and the *P* value following the permutation tests (i.e., permuted *P*). We found a significant negative correlation between the synchronization and complexity levels of the dyads, *r* = − 0.27, *P* = 0.007, permuted *P* = 0.003, 95% CI = [− 0.08, − 0.44], *BF*_10_ = 4.252, and between their synchronization and novelty levels, *r* = − 0.32, *P* = 0.001, permuted *P* < 0.001, 95% CI = [− 0.48, − 0.13], *BF*_10_ = 21.88 (Fig. 3a,b). This supports the notion that in more complex and novel interactions, it is harder to get into sync. These results suggest that humans do not only seek to be in sync with others, even when they are instructed to do so, but rather they seek to avoid predictable and repetitive interactions. They strive to be synchronized in an interesting, complex, and novel manner. Notably, novelty was found to be significantly positively correlated with complexity, *r* = 0.39, *P* < 0.001, permuted *P* < 0.001, 95% CI = [0.21, 0.54], *BF*_10_ = 344.21 (see Fig. 3c and Supplementary Material Fig. 1 for the permutation tests).

### Synchronization and complexity play a role in liking

**Figure 4:**
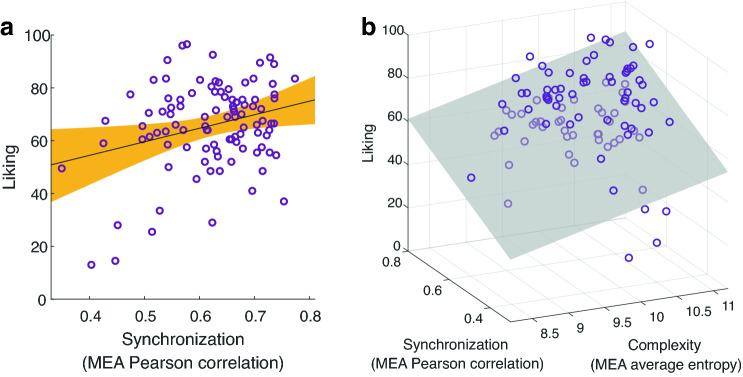
Predicting liking by dyadic movement features. (**a**) Synchronization level of the Z-scored MEA signals significantly predicted liking, *F*(1, 98) = 8.28, *P* = 0.005, permuted *P* = 0.005, *R*^2^ = .08, BF_10_ = 7.67. Each circle represents a dyad, the black line is the linear regression line, and the orange area marks the confidence interval around the slope of a regression line. (**b**) Multiple linear regression model including both synchronization and complexity significantly improved the model predictions, this model predicted 14.9% of the variance in liking, *R*^2^ = .149, *F*(2, 97) = 8.49, *P* < 0.001, permuted *P* < 0.001, *BF*_10_ = 66.54. Each circle represents a dyad in the 3D space and the gray plane marks the regression surface that was fitted by the model.

We used multiple linear regression models as well as Bayesian regression models to predict dyads' liking using synchronization, complexity, and novelty (see “Methods” section for more details). The dyadic liking was calculated as the average reported liking of the two players^8,11,22^ (see “Methods” section for more details). A model that included only the synchronization level predicted 7.8% of variance in liking, *F*(1, 98) = 8.28, *P* = 0.005, permuted *P* = 0.005, *R*^2^ = 0.078, *BF*_10_ = 7.67 (see Fig. 4 and Supplementary Material Fig. 2a). This result is in line with previous studies^6,8,9^, showing the linkage between synchronization and liking. Including complexity level into the model significantly improved it, explaining almost twice the variance of the former model. Accordingly, the linear model with both synchronization and complexity as predictors, predicted 14.9% of the variance in liking, *F*(2, 97) = 8.49, *P* < 0.001, permuted *P* < 0.001, *BF*_10_ = 66.54, with a positive correlation between synchronization level and liking, *β* = 0.35, *t*(97) = 3.63, *P* < 0.001, permuted *P* < 0.001, *BF*_*inclusion*_ = 47.97, and between complexity and liking, *β* = 0.28, *t*(97) = 2.85, *P* = 0.005, permuted *P* = 0.003, *BF*_*inclusion*_ = 13.86 (see Fig. 4b and Supplementary Material Fig. 2b–d). Notably, adding complexity level to the model increased the predictive power of synchronization, specifically in its Beta weight. This indicates that adding complexity to the model suppressed the irrelevant variability of synchronization and thus further improved the model. Introducing novelty to the model did not significantly improve it, and predicted an additional variance of 0.5%, *P* = 0.456, *BF*_10_ = 0.37. This result suggests that although the average novelty during the game is related to the synchronization level and to the complexity of the interaction, it does not provide added value in creating affiliation towards others. Following the comparisons between the models, as demonstrated in Fig. 4b (and in Supplementary Material Table 1), the best linear model out of the three was:

$${\text{Liking}} = - {59}.{33} + {64}.{73 } \times {\text{ Synchronization}} + {8}.{38 } \times {\text{ Complexity}}$$

Similar results were also found using maximal cross-correlation as the synchronization measure (see Supplementary Material Fig. 3) and when modeling the maximum liking or the minimum liking within each dyad, instead of the averaged liking (see Supplementary Material Figs. 5, 6). Moreover, we conducted a stepwise multiple linear regression on all available independent measures (see Supplementary Material for further details) to evaluate the effect of the order of the variables that were entered to the regression model. This analysis yielded almost identical results, therefore we are presenting here the results of the multiple linear regression, which is a more prominent method of analysis. Taken together, we found converging evidence that individuals tend to like others when their interactions are more complex and unpredictable, rather than simple and highly predictable.

### Dynamics of synchronization, complexity and novelty in dyads with high and low liking

**Figure 5:**
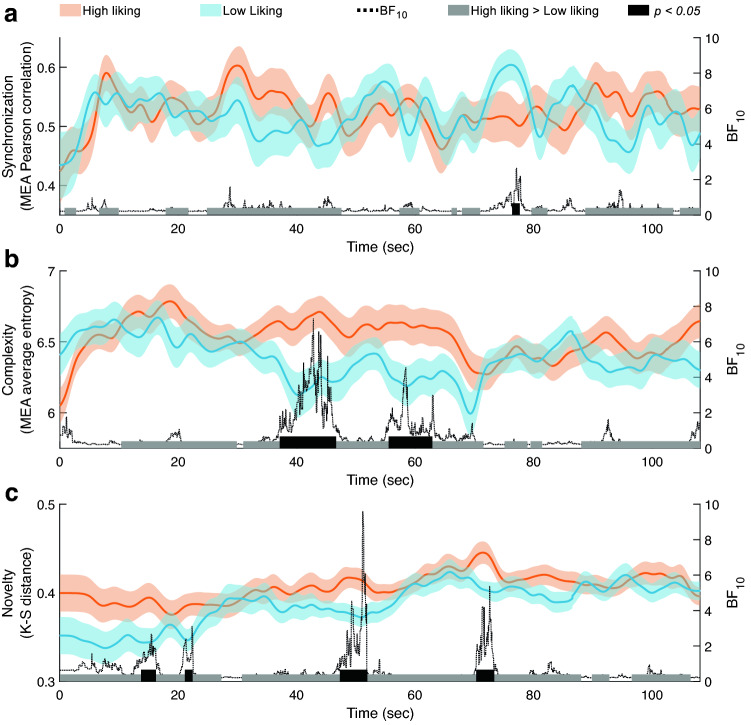
The dyadic movement measures along the interaction duration for high and low-liking. The orange and blue lines denote the average value of the high- and low-liking groups respectively, along the time-points of the Mirror Game. The shaded orange and blue marks denote the *SE*s of the high- and low-liking groups, respectively. The gray marks at the bottom of each panel denote time-points in which the high-liking group had a higher value than the low-liking group. The black marks denote time-points in which there was a significant difference between the liking groups. The black dashed lines depict two-tailed Bayes factors (*BF*_10_). (**a**) Synchronization was higher in the high-liking group than in the low-liking group for 55.87% of the game duration, binomial sign test *P* < 0.001. (**b**) Complexity was higher in the high-liking group than in the low-liking group for 79.62% out of the game duration, binomial sign test *P* < 0.001. (**c**) Novelty was higher in the high-liking group during 90.16% of the game, binomial sign test *P* < 0.001.

We were interested in testing the relationship between the dynamics of dyads' movements during the interaction and liking. To examine whether different patterns characterize high and low liking along the time domain, we used a median split^24,39,40^ that divided the data into low- and high-liking groups. Next, we compared the dynamics of synchronization, complexity, and novelty between the high-liking and low-liking groups, time-point by time-point, after dividing each game into movement segments^20^ (see “Methods” section for more details). As for synchronization, in 55.87% of the total game duration, the high-liking group moved in a more synchronized manner than the low-liking group, as marked in gray (Fig. 5a). Binomial sign test shows that this result was significantly above chance level (i.e. 50%), binomial *P* < 0.001. However, the difference between the two groups in a given time-point was significant (*P* < 0.05) for only 1.2% of the total game duration, in which the high-liking group was less synchronized than the low-liking group. Two-tailed Bayesian analysis showed no substantial evidence (*BF*_10_ < 3) that the high-liking group is less synchronized than the low-liking group. Similar results were also found using maximal cross-correlation as the synchronization measure (see Supplementary Material Fig. 7).

Regarding complexity (Fig. 5b), during 79.62% of the game duration, the movements of the high-liking group were more complex than those of the low-liking group, as marked in gray, which is significantly above chance level, as shown by a binomial sign test, *P* < 0.001. Furthermore, the difference between the high- and low-liking groups in a given time-point was significant for 15.66% of the duration of the game—the high-liking group moved in a more complex manner than the low-liking group. Finally, this was substantially supported by the Bayesian analysis in 4.01% of the Mirror Game duration.

In terms of novelty (Fig. 5c), unlike in the results of the multiple linear regression model, which did not take into account the time domain, in 90.16% of the game duration the high-liking group moved in a more novel manner than the low-liking group, as marked in gray, binomial sign test *P* < 0.001. Moreover, the difference between the high- and low-liking groups in a given time-point was significant for 10.7% of the game—the high-liking group was more novel than the low-liking group. Using Bayesian analysis, this was substantially supported in 1.94% of the duration of the Mirror Game.

As shown in Fig. 5c, it seems that novelty increased over time, both in the low- and the high-liking groups, but more prominently in the low-liking group. To test this, we performed a post-hoc correlation analysis and found that, indeed, the novelty significantly increased over time in the low-liking group, *r* = 0.82, *P* = 0.001, 95% CI = [0.70, 0.89], *BF*_10_ > 1**,**000, and in the high-liking group, *r* = 0.67, *P* = 0.001, 95% CI = [0.48, 0.80], *BF*_10_ > 1**,**000. Remarkably, a comparison between these Pearson coefficients showed that the increase in novelty over time of the low-liking group was significantly larger than that of the high-liking group, *z*(96) = 1.69, *P* = 0.045. This was also demonstrated by the slopes of the fitted linear regression lines: Novelty_low-liking_ = 6.34 × 10^–4^ × Time (s) + 0.35, Novelty_high-liking_ = 3.29 × 10^–4^ × Time (s) + 0.39. Notably, although the novelty of the high-liking group increased more slowly over time, the level of novelty in this group was relatively high right from the beginning of the Mirror Game, suggesting that people who like each other tend to create novel interactions from the very beginning, and then gradually increase the novelty level.

## Discussion

In this study we demonstrated that in addition to synchronization, complexity and novelty play a role in generating positive interactions. We found that synchronized participants who performed more complex and novel movements while playing the Mirror Game, liked each other more. Our results suggest that humans seek complexity and novelty, even when getting into sync will be more difficult.

In line with previous studies demonstrating that movement synchronization between individuals increases the positivity of social interactions^5,6^, we found that elevated synchronization during the Mirror Game was associated with elevated mutual liking (Fig. 4, 5a). Interpersonal synchrony may signal social proximity or similarity^15^ and plays an important role in social cohesion^16,48^. It was suggested that in small groups such as dyads, greater attention to the movements of each other and synchronized movements lead to a blurring between the self and the other^10,49^, or to put it differently, integration of self and other^44^. In turn, this increases the feeling of 'being in the zone' which is also known as togetherness^10,17,19,20^. Moreover, it was suggested that being synchronized is rewarding because it is an effective way to understand one’s interaction partner^15^. Consequently, such an alignment may facilitate communication^50^.

When playing the Mirror Game, the only instruction the dyads received was “to move their hands with as much coordination as possible”, i.e., to mirror each other. It is easier to be in sync when the movements are more predictable^25–27^, simple and repetitive. Although it made it harder for the participants to meet the single requirement of the experiment, in almost all of the Mirror Games (95%), both players influenced each other's motion, as was demonstrated by the GCA results. This occurs when two participants reciprocally adjust their ongoing rhythms as a result of an interaction, serving as a reliable marker for mutual sharing of information^51,52^. This is in contrast to the leader–follower type of interaction, which contains only a one directional information flow. In addition, almost all the dyads (99%) chose to perform unpredictable, novel movements, rather than merely performing synchronized movements. This is despite the fact that predictability facilitates synchronization^25–27^, and therefore it was harder to get into sync than when moving in a highly repetitive and predictable manner. Accordingly, the results suggest that participants preferred to decrease their potential synchronization, in order to increase the mutual interest and experience a better interaction. Moreover, though independent in principle, there was a positive relationship between complex and novel movements, suggesting that participants preferred to be unpredictable both in terms of what information may be known from time *t* on time *t* + 1 and in terms of repetitivity, and thus made the interactions more interesting. Altogether, the results imply that strong relationships between mutual influence and positive interactions^53^ and between elevated interest and positive interaction^54^ are fundamental for human beings.

Taking into account the whole interaction, we found that the synchronization and complexity of movements predicted liking more accurately than only synchronization of movements (Fig. 4). Adding complexity to the model seems to suppress the irrelevant variability of synchronization and further improved the prediction of liking. In addition, synchronized and complex movements were negatively correlated (Fig. 3a). This finding implies that liking relies on a tradeoff between the synchronization and the complexity of an interaction. In other words, the results support the notion that on one hand people seek predictable interactions which facilitate synchronization, and in turn are rewarding^15^, but on the other hand, people seek interesting and challenging interactions. Complex interactions are by definition less predictable and comprise a high level of information transformation during communication (as mentioned regarding the definition of entropy). Thus, an optimal balance between the two may maximize the reward and the quality of the interaction.

In contrast to the complexity of movements, when adding the average novelty of the interaction to the model, there was no improvement in predicting liking. Possibly, this is because novelty seeking is a fundamental drive^29,34,35^ and, accordingly, may be a basic need in social interaction. Subsequently, it is plausible that during the Mirror Game as a whole, the dyads played in a novel manner, regardless of the level of liking. In 99% of the cases, the movements of the dyads were more novel than just repetitively moving their hands up and down, even though there was no requirement to move in a novel manner, which makes it harder to be in sync. Moreover, novelty increased over time, and this trend was even more pronounced in the low-liking group than in the high-liking group, which was relatively novel from the beginning of the game.

Our results showed that the dynamics of synchronization along the Mirror Game time-points, unlike the synchronization of the dyads′ movement across the entire game, was only mo derately related to liking (Fig. 5a). This discrepa ncy between the game as a whole and the dynamics on a movement-to-movement basis suggests that, in this case, the whole is greater than its parts, and to identify differences in synchronization, one has to consider the interaction as a whole. During the Mirror Game, people got in and out of sync, in accordance with previous findings^33,55^. There were fluctuations in the level of synchronization along the movement segments, but overall people who liked each other were more synchronized. This is in line with the suggestion that interacting individuals are dynamically coupled rather than simply aligned. In teractions are dynamic states which involve continuous mutual adaptation during which complementary behavior develops^33^. This notion was recently demonstrated in the context of pupil dilation synchronization during a conversation, which was found to fluctuate according to the shared attention pattern^36^. Such continuous mutual adaptation along the time domain generates synchrony and, in doing so, promotes shared understanding^56^. We suggest that in order to increase the complexity and novelty of the interaction, humans are willing to sacrifice synchronization to some extent, as a part of mutual adaptation. Together, complexity and novelty make an interaction interesting and meaningful—an interaction that one would want, unlike a highly synchronized, but very predictable, simple and repetitive, boring interaction. This notion was supported by the positive relationship between the complexity and novelty of movements along the time domain, to liking. We suggest that when people feel confident enough in an interaction, they are willing to take the risk of losing synchronization, while trying to increase the level of complexity and novelty in order to make the interaction even more satisfying. Then, they restore the synchronization and subsequently balance the two back and forth.

To summarize, in addition to the canonical view that the interaction quality relies on the link between synchronization and prosocial emotions^19,57^, we argue that complexity and novelty are crucial and even indispensable factors in human social interactions. Accordingly, we propose a new framework in which optimizing the interaction quality requires a delicate balance between being synchronized and generating challenging and interesting interaction.

## Methods

### Participants

Twenty-six naïve Israeli females participated in this study, in three round-robin sessions^58^, i.e., each participant played the game with all of the other participants in the same session. Thus, there were 15 dyads in the first session, 45 dyads in the second session and 45 dyads in the third session. We recruited only females since the link between movement synchrony and affect has been found to be stronger in female dyads than in male dyads^45^. We recruited the participants using social media, and to validate that they do not know each other we conducted preliminary phone interviews. All participants provided a written informed consent to participate in the experiment, which was approved by the ethics committee of the Neurobiology department at Weizmann Institute of Science. This study was carried out in accordance with approved guidelines. All participants received monetary reward and signed a consent in which they allowed us to analyze their data, including their videos. Among the participants in this study, 10 have originally participated in another study that is yet to be published. Importantly, its analyses are not related to this study. The experiment took place over three different sessions: the first session included six participants (*mean* age = 26.83, *SD* = 4.21 years); the second session included ten participants (*mean* age = 25.6, *SD* = 3.1 years); the third session included ten participants (*mean* age = 26, *SD* = 3.46 years). In each round of the round-robin design, participants were randomly assigned partners and played the Mirror Game (see details below) for about two minutes. The duration of the Mirror Game ranged between 109.81 to 130.56 s (*mean* duration = 119.84, *SD* = 2.5). A sound indicated when to start the Mirror Game and when to stop it after the time is up. However, during the video processing we noticed that the dyads tended to start in a delay of a few seconds and to stop in a similar delay. In accordance with that, to include the whole Mirror Game duration, from the actual starting point until the actual stopping point, the dyads varied in the time duration they played. We excluded one dyad from the analyses, due to lack of movement during the game. Following that, a total of 104 dyads were included in the motion analyses. The sample size is in accordance with previous studies about the relationship between movement synchronization and rapport (e.g., Vacharkulksemsuk et al.^11^). According to previous findings the strength of the correlation between movement synchronization and liking is approximately equal to *r* = 0*.*4 in a simple tapping task (in experiment 1, *r* = 0.39 and in experiment 2, *r* = 0.40)^10^. However, because of the complexity of the Mirror Game task relative to a tapping task, we expected lower correlation coefficient ranging between *r* = 0.25 and *r* = 0.3. Using G-power, we ran power analysis for this range of expected effect sizes, which corresponds to a sample size of 85–123 dyads.

### Experimental design

Participants were assigned to pairs and played the full-body Mirror Game^37^, in which they were only asked to freely move their hands with as much coordination as possible, without talking, for two minutes**,** while keeping their legs in place. There were no instructions regarding the direction of the movement or regarding the leader and the follower (see Fig. 1a, both participants in the dyad shown in the figure signed an informed consent to publish the image in an online open access publication). Each dyad played the game in a separate room. During the game, participants stood at a distance of 50 cm apart, which was marked on the floor. Participants were not allowed to speak with each other during the entire experiment; thus only non-verbal factors influenced the impression formation. After each dyadic interaction, both players indicated on a visual analog scale ranging from 0 to 100 how much they liked their partner. These subjective reports were done via computers in a class with distributed computers, so no one could see the others' screen. Throughout each game, participants were filmed by a hidden camera alarm clock, which filmed them in 29.97 frames per seconds (FPS) and was set on a table in each of the five rooms used for the experiment, at a distance of 159 cm from where the players stood. After we completed the data collection, we used the video recordings to verify that all participants followed the instructions and moved during the whole game, mirroring each other without talking. In addition, we used the video recordings to analyze participants' movement.

### Motion energy analysis

We used Motion energy analysis (MEA) to quantify each player's motion throughout the Mirror Game^38^. Motion energy was defined as frame-by-frame differences in pixels color between consecutive video-frames^21,59^. Since, throughout the recordings, both camera position and lighting conditions were kept constant, any frame-by-frame changes indicated body motion of the respective player and not of its surroundings. Following Ramseyer and Tschacher^21^, we performed video-noise reduction using automatic detectors for time-series of raw pixel-change. The MEA signal of each player was transformed to Z-scores to scale the final values^21,24,45^ and thus account for differences in the players' height and hand size (Fig. 2). We performed the analyses both at the level of the whole game and along movement segments^20^.

### Granger causality analysis (GCA)

GCA is a method that uses autoregressive models to measure the causal relationship (i.e., Granger causality) between two time series^41^. This is done by testing whether one time series can predict the other above and beyond the auto-prediction of this time series, and vice versa^41^. In each dyad, we computed the pairwise-conditional causalities of each participant's movement to that of the other participant. This allowed us to examine whether one member is the leader and the other is the follower or both members are interchangeably leaders and followers, such that each member’s movement influenced the other’s movement.

### Segmentation into movements

To divide the Z-scored MEA vectors of each dyad into movement segments, first the signals were smoothed using moving average to eliminate short-term trends and to improve signal to noise ratio^21,45^. This was done using the function ‘smoothdata’ in MATLAB 2020a. The optimal time-window for the moving average was heuristically evaluated using the default ‘SmoothingFactor’ parameter in the function ‘smoothdata’. Next, we searched for local minima within the signal of each dyad member, i.e., stopping and deceleration points, using the function ‘findpeaks’. Each minimum within the signals had to meet the following conditions: (1) The value in both signals was smaller than zero (i.e., the movement velocity was smaller than the average movement velocity, for example see the first square from the left in Supplementary Material Fig. 8); (2) To be defined as an actual minimum point (rather than a local fluctuation), a minimum point must be in a distance of at least the length of the optimal time-window from the previous minimum point in the signal of this dyad member (for example, see the forth square from the left in Supplementary Material Fig. 8); (3) The signal amplitude (estimated velocity) must change by at least 0.1 SDs between two consecutive minimum points (to compensate for fluctuations of the signal around the minimum point, for example see the third square from the left in Supplementary Material Fig. 8). After finding the minimum points in the signal of each participant in each dyad, we searched for the minimum points with the smallest time differences between the participants in each dyad and defined them as shared minimum points (for example see the second square from the left in Supplementary Material Fig. 8). Two consecutive shared minimum points had to be in a distance of at least the length of the optimal moving average window. After finding the shared minimum points, to set a starting point and an ending point for each movement, we averaged the time indices of the shared minimum points in each dyad’s vectors (as shown by the gray vertical lines in Fig. 1b, demonstrating a representative movement segmentation of the Mirror-Game time course). We provided the code for the segmentation into movements algorithm in the Supplementary Material.

### Measuring movement novelty

We developed a new method for quantifying dyadic novelty—how different (not-repetitive) each movement segment was compared to the previous segments, and accordingly, what is the overall dyadic novelty. Using the Kolmogorov–Smirnov distance (K-S distance) test^60^, we quantified the distance between the empirical distribution functions of the Z-scored MEA of each segment to each of the previous segments. Each test consists of two segments at a time. Notice that the two-sample Kolmogorov–Smirnov test, which is used to test whether two samples come from the same distribution, is well-suited for our case, as it is sensitive to differences in both location and shape of the empirical cumulative distribution functions (CDFs) of each two segments. In general, the distance between empirical CDFs is small when the distributions are similar (it is zero when they are identical) and close to one when they are very different.

For each member within each dyad, we first compared the first and the second movement segments using the K-S distance test. Then, for each dyad, we averaged between the two members’ statists of the K-S distance test, in order to get a single dyadic statist for the movement novelty until the second movement segment. This served as the dyadic novelty score of the second segment. Since there are no segments before the first one, we also assigned it as the novelty score of the first segment. Next, for each dyad member, we compared the third segment to each of the previous segments: we compared the third segment and the second segment, and the third segment and the first segment. The average statist of the K-S distance tests of the two comparisons, averaged also within the dyad members, served as the dyadic novelty score of the third segment. Similarly, we compared the fourth segment and each of the previous segments, and averaged for each member the three resulting statists of the K-S distance tests. We then also averaged between the two players within each dyad, to get the dyadic novelty score for the fourth segment. This procedure was done for each dyad up to segment N. Thus, each segment was assigned a novelty score, which enabled tracking the novelty changes along the time domain. In order to have also a single novelty score for each dyad, we averaged the novelty scores of all the segments (without the first one, which got the same value as the second segment) within each dyad.

To establish a reference point and get a sense of the dyads’ movement novelty, we compared it to an example of low-novelty movement and an example of high-novelty movement. This was done by filming a volunteer (who was not a part of the Mirror Game cohort) twice: once while periodically moving their hands up and down for two minutes in a highly repetitive manner, and once while moving their hands in a highly innovative way, by varying their speed and angles, trying not to repeat any movement pattern, for two minutes. This was done using the exact same setup used in the Mirror Game experiment. We calculated the Z-scored MEA of these two videos and then calculated their novelty (using the K-S distance). The low-novelty (LN) movement is shown in the upper panel of Fig. 2a, and demonstrating a highly repetitive signal, and the high-novelty (HN) movement is shown in the bottom panel of Fig. 2a, and demonstrating a diverse and non-repetitive signal.

In addition to objectively measuring the novelty of the movements, a judge watched each video of the Mirror Game, and was requested to decide whether the players had moved differently than simply moving their hands up and down repeatedly throughout the whole interaction (or otherwise in a highly repetitive and thus predictive manner, e.g. from side to side or outwards and then inwards).

### Measuring movement synchronization

In accordance with previous studies, we used Pearson correlation to measure the synchronization level of dyads^19^, and thus measured the degree of accelerating or decelerating at the same time. A limitation of Pearson correlation is that it does not calculate correlations with time shifts between the signals, but only the correlation in zero lag. Thus, we also used cross-correlation^43^ as an alternative measure for synchronization which is widely used in the literature. This allowed us to define the synchronization as the synchronization in the time-shift that shows the highest similarity between the two signals, i.e., the maximal cross-correlation (see Supplementary Material Fig. 7 for the results of the analyses using the maximal cross-correlation).

### Measuring movement complexity

We used Shannon entropy^46^—the average level of information—as a measure of the complexity of dyad’s movements. Although movement is a continuous signal, we obtained a sample every 0.033 s, so our data were discrete. The entropy of a discrete variable $X$ that can take values in the set $A$ is defined as:

$$H\left(X\right)=-{\sum }_{x\in A}p\left(x\right)\mathrm{log}p\left(x\right).$$

To calculate the entropy of each player's Z-scored MEA signal, we first calculated the frequency of each unique MEA value per sample, and then calculated the entropy using the Entropy function in MATLAB 2020a. Next, we averaged the entropies of the two participants in each dyad as the relative entropy differences within each dyad were very small (see Supplementary Material Fig. 4a). This was done in order to get a single measure of dyadic entropy, which represents the dyadic average level of movement information. Communication is said to occur when information flow from one location to another and cause a change in the receiver^46^. Information theory, the theoretical framework underpinning entropy, attributes higher levels of information to more unpredictable distributions. In other words, when an interaction is less predictable it is more complex^25,47^. Therefore, unpredictable interactions are more informative and complex than predictable ones, and hence also more interesting.

### Measuring liking

To estimate participants’ liking, we calculated a dyadic score of likeability per each Mirror Game, defined as the average reported liking of the two players. Following previous studies in the context of social interactions, we chose the average liking ratings within each dyad as a dyadic measure for liking^8,11,22^. In addition, we found that the liking differences within each dyad were relatively small (see Supplementary Material Fig. 4c).

We wanted to test whether movement synchronization, complexity, and novelty predict participants’ liking. To do so, we used multiple linear regression models and calculated the explained variance of liking by synchronization solely, synchronization together with complexity, and synchronization together with complexity and novelty. A total of three models were tested using JASP version 0.14.1.0 software. In addition, we calculated the Pearson correlation between synchronization, complexity, novelty and mutual liking. We excluded four dyads from the analyses, who were farther than 3 SDs from the average (two were outliers due to their complexity levels, and two due to their liking ratings). In total, the analyses were performed on 100 dyads.

### Movement along the time domain

To test whether dyads' movement along the time domain during the interaction reflected liking, we divided the dyads according to the median^24,39,40^ into two groups: high-liking half and low-liking half, each comprising 50 dyads. We then compared the synchronization, complexity, and novelty along the time domain between the two groups. Low- and high-liking signals were smoothed using a moving average of four seconds (see Fig. 5), which was the average segment length, to improve signal to noise ratio.

### Permutation tests

In order to take into account the fact that each participant was a member of more than one dyad, we added permutation tests (see Supplementary Material). In these tests, we compared in each analysis the actual statistic value to a null distribution. For example, to test whether the correlation between synchronization and complexity was significant, we generated a null distribution by randomly shuffling the dyadic labels of the average complexity levels, such that each dyadic synchronization was paired with another dyad’s average complexity. We did so 10,000 times and then correlated the permuted complexity and the original (non-permuted) synchronization level of the dyads. Accordingly, we preserved the original dyadic values that were obtained in the round-robin sessions, and generated a null distribution by decoupling the original dyadic synchronization and complexity. Next, we assessed the significance level by testing where the real correlation coefficient fall on the null distribution. We performed the same procedure to test the significance of the synchronization and novelty correlation coefficient, and the complexity and novelty correlation coefficient. A similar procedure was performed to test the significance level of the liking models, generating *F* and *t* null distributions.

### Bayesian analysis

We have provided a complementary measure to the standard *P* values^61^, using Bayes factors (*BF*) calculated with the JASP software; priors were set according to default JASP priors^62^. The interpretation of the Bayes factors was according to the following scale: Bayes factors between 3 and 10 provide substantial evidence against H_0_; Bayes factors between 10 and 100 provide strong evidence against H_0_; and Bayes factors above 100 provide decisive evidence against H_0_^63^. The Bayes factors calculated for the predictors of the regression models (*β*s' weights) were inclusion Bayes factors, denoting the change in the prior to posterior probability inclusion odds when including the predictor^64^.

## Supplementary Information

Supplementary Information.

## Data Availability

The code for the analysis of the segmentation into movements is available in the Supplementary Material. The data that support the findings in this study are available at https://osf.io/6pbgf/?view_only=b02268caee0a4ee4ae728d14bcbb5a65.
