# Supplementary material for: Liking as a balance between synchronization, complexity and novelty
Source: Sci Rep. 2022 Feb 24;12:3181. doi: 10.1038/s41598-022-06610-z (PMC8873358; doi:10.1038/s41598-022-06610-z)
Supplement: Supplementary file 1 — Supplementary Information. [file 41598_2022_6610_MOESM1_ESM.pdf]

Supplementary Material

Segmentation. The segmented mirror games durations were between 84.62 to 127.56 seconds. The time window analysis was performed for a duration of 108.2 seconds, in order to include the entire time series of the vast majority of the games (only four games ended before this point: after 84.62 sec, 100.57 sec, 106.2 sec and 107.74 sec. These dyads are included in the statistics until the mentioned ending point of each). Notice that the last segment is defined as the last movement between two stopping or decelerating points, and hence the partial movement after it is not considered as a movement segment.

Permutation Tests. In order to take into account the fact that each participant was a member of more than one dyad, we added permutation tests. In these tests, we compared in each analysis the actual statistic value to a null distribution. For example, to test whether the correlation between synchronization and complexity was significant, we generated a null distribution by randomly shuffling the dyadic labels of the average complexity levels, such that each dyadic synchronization was paired with another dyad's average complexity. We did so 10,000 times and then correlated the permuted complexity and the original (non-permuted) synchronization level of the dyads. Accordingly, we preserved the original dyadic values that were obtained in the round robin sessions, and generated a null distribution by decoupling the original dyadic synchronization and complexity. Next, we assessed the significance level by testing where the real correlation coefficients fall on the null distribution. We performed the same procedure to test the significance of the synchronization and novelty correlation coefficient, and the complexity and novelty correlation coefficient. As shown in Supplementary Material Fig. 1, the results of the permutation tests indicate that each of the three real correlations falls within the extreme 5% of the null distributions, and accordingly was significant (synchronization vs. complexity P = .003, synchronization vs. novelty P = < .001 and complexity vs. novelty P < .001). This indicates that the original results showing a negative relationship between synchronization and complexity and between synchronization and novelty, and a positive relationship between complexity and novelty, were not a result of the nature of the round robin design.

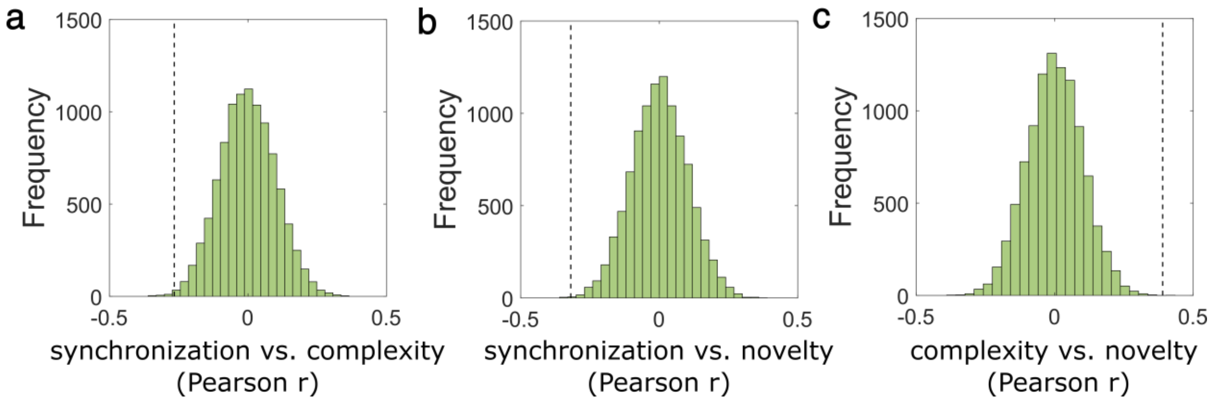

Supplementary Material Fig. 1. Results of permutation testing for Pearson correlations between the different movement measures. Histograms depict the distribution of Pearson *r* coefficients across 10,000 permutations of the data set, in which (a) the complexity of each dyad was randomly shuffled and then correlated with the synchronization level of the dyads, (b) the novelty of each dyad was randomly shuffled and then correlated with the synchronization level of the dyads and (c) the complexity of each dyad was randomly shuffled and then correlated with the novelty of the dyads. The black dashed lines depict the actual Pearson *r* of each correlation analysis (i.e., based on the non-permuted data). Results of these permutation tests indicate that synchronization and complexity are significantly negatively correlated (P = .003), that synchronization and novelty are significantly negatively correlated (P < .001) and that complexity and novelty are significantly positively correlated (P < .001).

To generate the null distributions for the liking models, the labels of the average liking ratings of each dyad were randomly shuffled between dyads, such that each dyadic liking was paired with another dyad's synchronization, complexity and novelty. We did so 10,000 times. Then, to assess the significance level, we ran the linear regression analyses with the permuted liking ratings, obtaining a null distribution for each model. The results of the permutation tests indicated that synchronization significantly predicted average liking (permuted P = .005, see Supplementary Material Fig. 2a) and also that synchronization and complexity significantly predicted the average liking (permuted P < .001, see Supplementary Material Fig. 2b). As shown in Supplementary Material Fig. 2c-d, when predicting the average liking by synchronization and complexity, the results of the permutation tests for the t statistic of synchronization β and for t statistic of complexity β were also significant (permuted P < .001 and permuted P = .003, respectively).

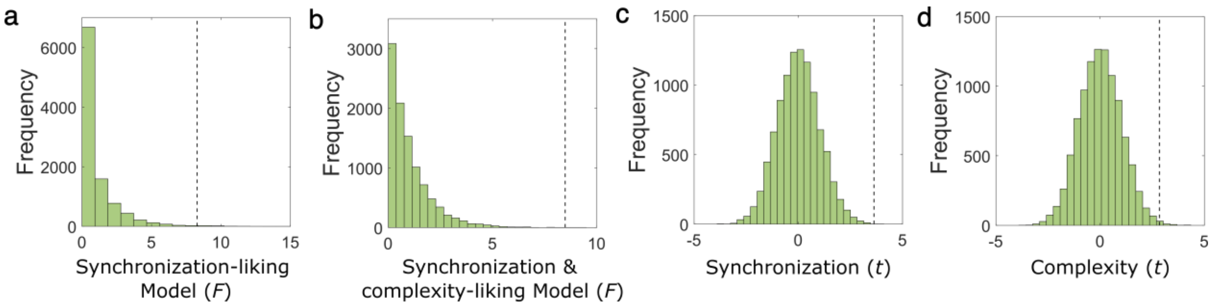

Supplementary Material Fig. 2. Results of permutation testing for average liking models. Results of permutation tests for predicting the average liking by (a) synchronization and for (b) predicting the average liking by synchronization and complexity. Histograms depict the distribution of *F* statistics across 10,000 permutations of the data set, in which the liking ratings of each dyad were randomly shuffled. Black dashed lines depict the actual *F* statistic of each model (i.e., based on the non-permuted data). Results of these permutation tests indicate that synchronization predicted the average liking more than would be expected by chance ($P < .001$), and that synchronization and complexity also predict the average liking above chance ($P = .005$). (c) Results of permutation tests for t statistic of synchronization β and (d) for t statistic of complexity β when predicting the average liking by synchronization and complexity. Histograms depict the distribution of *t* statistics across 10,000 permutations of the data set, in which the liking ratings of each dyad were randomly shuffled. Black dashed lines depict the actual *t* statistic of each model (i.e., based on the non-permuted data). Results of these permutation tests indicate that synchronization β and complexity β are above chance, ($P < .001$ and $P = .003$ respectively).

Measuring Synchronization by Maximal Cross-Correlation Instead of Pearson Coefficient Showed Similar Results. We used multiple linear regression models as well as Bayesian regression models in order to predict dyads' liking by level of synchronization, complexity and novelty. A model including only the level of synchronization predicted 6.1% of variance in liking, $F(1, 98) = 6.41$, $P = .013$, permuted $P = .015$, $R^2 = .061$, $BF_{10} = 3.464$ (Supplementary Material Fig. 3). Including the complexity level significantly improved the model and predicted an additional 6.1% of variance in liking. Accordingly, the linear model with both synchronization and the average entropy as predictors predicted 12.2% of the variance in liking, $F(2, 97) = 6.76$, $P = .002$, permuted $P = .002$, $BF_{10} = 17.303$, with a positive correlation between the level of synchronization and liking, $\beta = 0.30$, $t(97) = 3.137$, $P = 0.002$, permuted $P = .002$, $BF_{\text{inclusion}} = 12.976$ and complexity level and liking, $\beta = .254$, $t(97) = 2.60$, $P = .011$, permuted $P = .007$, $BF_{inclusion} = 5.924$ (Supplementary Material Fig. 3b). Introducing novelty to the model did not significantly improve it, and predicted an additional variance of 0.02%, $P = .620$, permuted $P = .315$, $BF_{10} = .337$. This result suggests that although the average novelty during the game is related to the synchronization level and the complexity of an interaction, it is not sufficient to create affiliation towards others. Following the comparisons between the models, as demonstrated in Supplementary Material Fig. 3b, the best linear model out of the three was:

$$\text{Liking} = -54.79 + 64.4 \times \text{Synchronization} + 7.77 \times \text{Complexity}$$

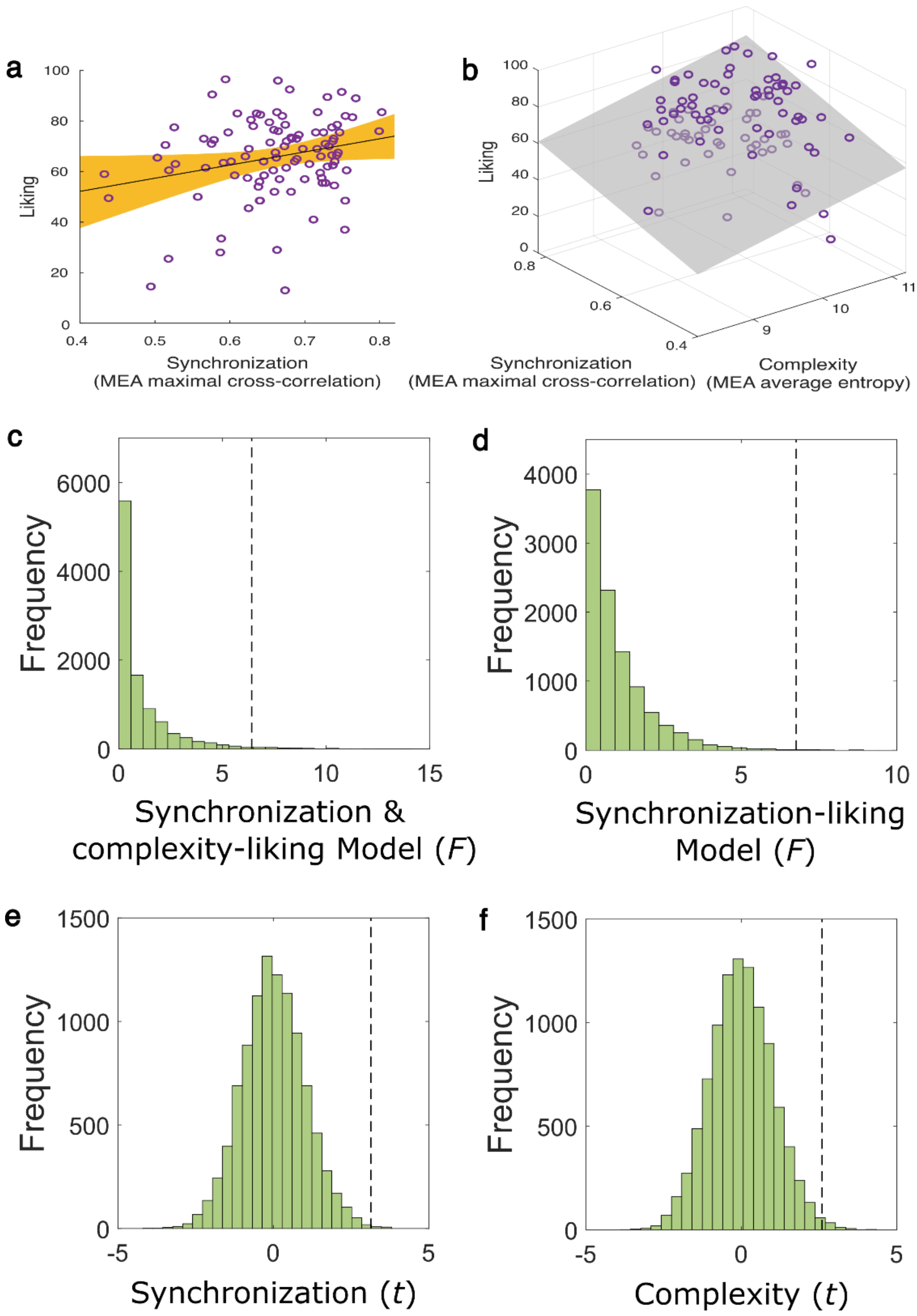

Supplementary Material Fig. 3. Predicting liking by dyadic movement features. (**a**) Synchronization level of the z-scored MEA signals significantly predicted the average liking, $F(1, 98) = 6.41$, $P = .015$, permuted $P = .015$, $R^2 = .061$, $BF_{10} = 3.46$). Each circle represents a dyad, the black line is the linear regression line, and the orange area marks the confidence interval around the slope of a regression line. (**b**) Multiple linear regression model including both synchronization and complexity significantly improved the model predictions (this model predicted 12.2% of the variance in liking, $R^2 = .122$, $F(2, 97) = 6.76$, $P = .002$, permuted $P = .002$, $BF_{10} = 17.30$). Each circle represents a dyad in the 3D space and the grey plane marks the regression surface that was fitted by the model.

Relative Differences within the Dyads. To estimate the relative differences, i.e., how large the difference between the dyad members was relative to the used scale, we calculated for each measure the absolute difference between the partners divided by the sum: $\frac{|x_1-x_2|}{|x_1|+|x_2|}$. By that we also obtained a normalized scale ranging from 0 (no difference) to 1 (the maximal possible difference) which is comparable between different measures.

As demonstrated in Supplementary Material Fig. 4, the distributions of the data show very small differences in the movement measures (complexity *mean* = .014, *SD* = .029, novelty *mean* = .041, *SD* = .070). Only 14 out of the 100 dyads differed from each other by more than one SD from the relative complexity difference mean (Supplementary Material Fig. 4a), and only 12 out of the 100 dyads differed from each other by more than one SD from the relative novelty difference mean (Supplementary Material Fig. 4b). In addition, the maximum relative difference in complexity was .091, and the maximum relative difference in novelty was .160. These results show that the complexity and the novelty of the movements within the dyad members were highly similar. Moreover, although higher than the movement measures' relative differences, the liking rating's relative differences were also quite small, with a *mean* of .179 and *SD* of .190, where 14 out of the 100 dyads differed from each other by more than one SD from the relative liking difference mean (Supplementary Material Fig. 4c). These results suggest that the differences in the liking ratings are relatively small.

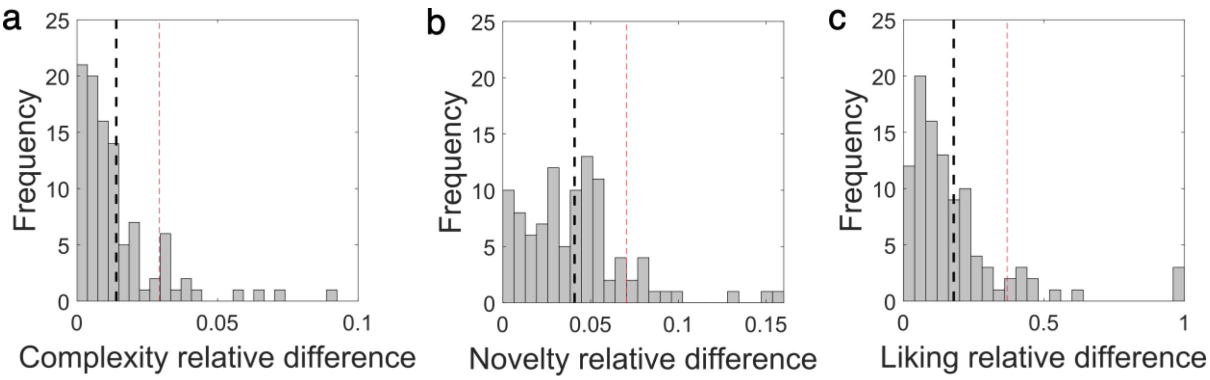

Supplementary Material Fig. 4. Histograms showing the relative differences of each measure within the dyad's members. (a) Complexity, (b) novelty and (c) liking. The black dashed lines depict the average relative difference of each measure and the red dashed lines depict one SD above the average relative difference of each measure.

Although the differences in the liking ratings within the dyads were relatively small, one may ask about the relationship between the dyadic movement features (i.e. synchronization, complexity and novelty) and the liking rating of each dyad member. More specifically, the more extreme partner within each dyad might bias the interaction. To investigate this, we used

multiple linear regression models as well as Bayesian regression models in order to predict the maximum and minimum liking within each dyad by the levels of synchronization, complexity, and novelty. Noticeably, as shown below, the results of the maximum liking models as well as the minimum liking models were very similar to the results of the average liking models.

First, we tested the models predicting the maximum liking within each dyad. Similar to the previous analyses, also here we used permutation tests to take into account that each participant was a member of more than one dyad. A model that included only the level of synchronization predicted 5.1% of the variance in maximum liking, $F(1, 98) = 5.25$, $P = .024$, permuted $P = .024$, $R^2 = .051$, $BF_{10} = 2.106$ (Supplementary Material Fig. 5a, 5c). Including the complexity level significantly improved the model and predicted an additional 4.770% of the variance in maximum liking. Accordingly, the linear model with both synchronization and complexity as predictors predicted 9.987% of the variance in maximum liking, $F(2, 97) = 5.310$, $P = .006$, permuted $P = .007$, $BF_{10} = 5.426$, with a positive correlation between the level of synchronization and maximum liking, $\beta = 0.286$, $t(97) = 2.859$, $P = 0.005$, permuted $P = .005$, $BF_{inclusion} = 5.029$ and complexity level and maximum liking, $\beta = .227$, $t(97) = 2.268$, $P = .026$, permuted $P = .016$, $BF_{inclusion} = 2.783$ (Supplementary Material Fig. 5b, 5d-f). As in our original analysis with the averaged liking ratings, introducing novelty to the model did not significantly improve it, and predicted an additional variance of 0.8%, $P = .355$, permuted $P = .176$, $BF_{10} = .877$. Following the comparisons between the maximum liking models, as demonstrated in Supplementary Material Fig. 5b, the best linear model out of the three was:

$$\text{Maximum liking} = -19.58 + 48.77 \times \text{Synchronization} + 6.39 \times \text{Complexity}$$

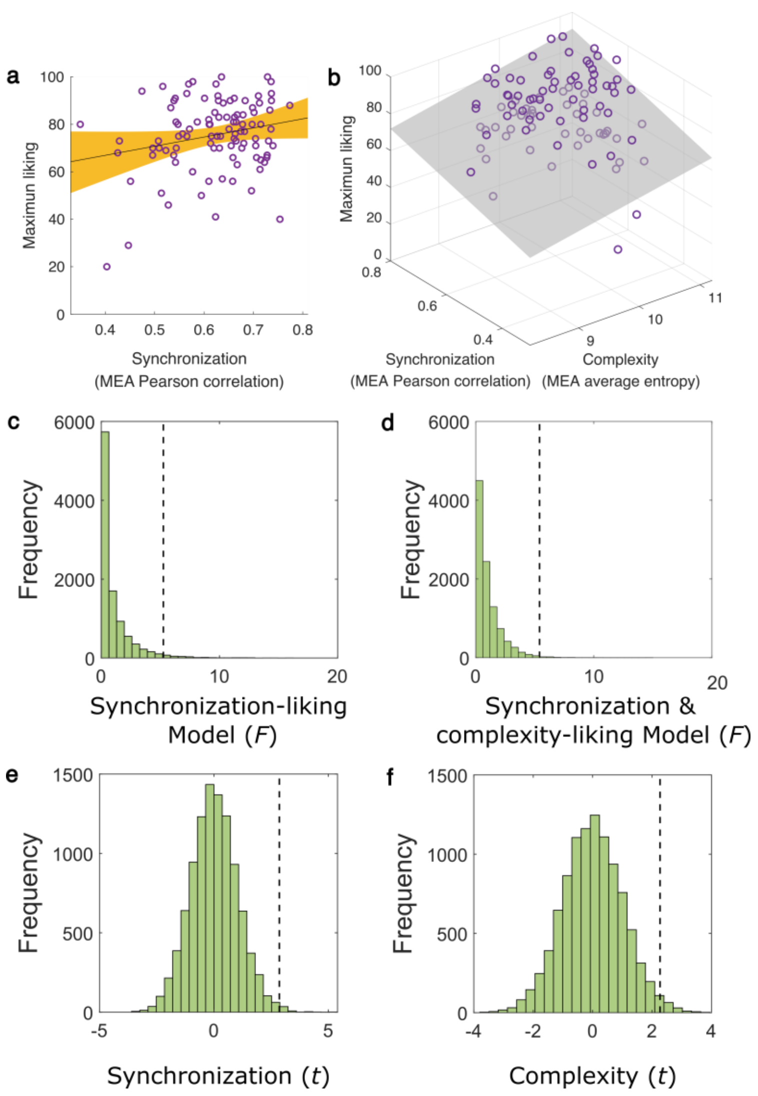

Supplementary Material Fig. 5. Predicting maximum liking by dyadic movement features. (a) Synchronization level of the z-scored MEA signals significantly predicts the maximum liking within each dyad, *F*(1, 98) = 5.25, *P* = .024, permuted *P* = .024, R^2 = .051, BF_{10} = 2.106. Each circle represents a dyad, the black line is the linear regression line, and the orange area marks the confidence interval around the slope of a regression line. (b) Multiple linear regression model including both synchronization and complexity significantly improved the model predictions, this model's predicted 9.87% of the variance in the maximum liking within each dyad, *F*(2, 97) = 5.31, *P* = .006, BF_{10} = 5.426. Each circle represents a dyad in the 3D space and the grey plane marks the regression surface that was fitted by the model. (c) Results of permutation tests for predicting the maximum liking by synchronization and for (d) predicting the maximum liking by synchronization and complexity. Histograms depict the distribution of *F* statistics across 10,000 permutations of the data set, in which the liking ratings of each dyad were randomly shuffled. Black dashed lines depict the actual *F* statistic of each model (i.e., based on the non-permuted data). Results of these permutation tests indicate that synchronization significantly predicted maximum liking (*P* = .024), and that synchronization and complexity also predict maximum liking above chance (*P* = .007). (e) Results of permutation tests for t statistic of synchronization *β* and (f) for t statistic of complexity *β* when predicting the maximum liking by synchronization and complexity. The histograms depict the distribution of *t* statistics across 10,000 permutations of the data set, in which the liking ratings of each dyad were randomly shuffled. Black dashed lines depict the actual *t* statistic of each model (i.e., based on the non-permuted data). Results of these permutation tests indicate that synchronization *β* and complexity *β* are above chance, (*P* = .005 and *P* = .016 respectively).

Next we ran the same analyses, but this time with the models predicting the minimum liking within each dyad. A model including only the level of synchronization predicted 8.3% of the variance in minimum liking, *F*(1, 98) = 8.82, *P* = .004, permuted *P* = .003, R^2 = .083, BF_{10} = 9.641 (Supplementary Material Fig. 6a, 6c)**.** Including also the complexity level significantly improved the model and predicted an additional 7.3% of the variance in minimum liking. Accordingly, the linear model with both synchronization and complexity as predictors predicted

15.6% of the variance in minimum liking, $F(2, 97) = 8.995$, $P < .001$, permuted $P = < .001$, $BF_{10} = 98.548$, with a positive correlation between the level of synchronization and minimum liking, $\beta = .362$, $t(97) = 3.746$, $P < .001$**,** permuted $P < .0.001$, $BF_{inclusion} = 69.343$ and complexity level and minimum liking, $\beta = .282$, $t(97) = 2.914$, $P = .004$, permuted $P = .003$, $BF_{inclusion} = 17.015$ (Supplementary Material Fig. 6b, 6d-f). Introducing novelty to the model did not significantly improve it, and predicted an additional variance of 0.3%, $P = .615$**,** permuted $P = .304$, $BF_{10} = .895$. This result suggests that although the average novelty during the game is related to the synchronization level and the complexity of an interaction, it is not sufficient to create affiliation towards others. Following the comparisons between the minimum liking models, as demonstrated in Supplementary Material Fig. 6b, the best linear model out of the three was:

$$\text{Minimum liking} = -99.901 + 80.696 \times \text{Synchronization} + 10.369 \times \text{Complexity}$$

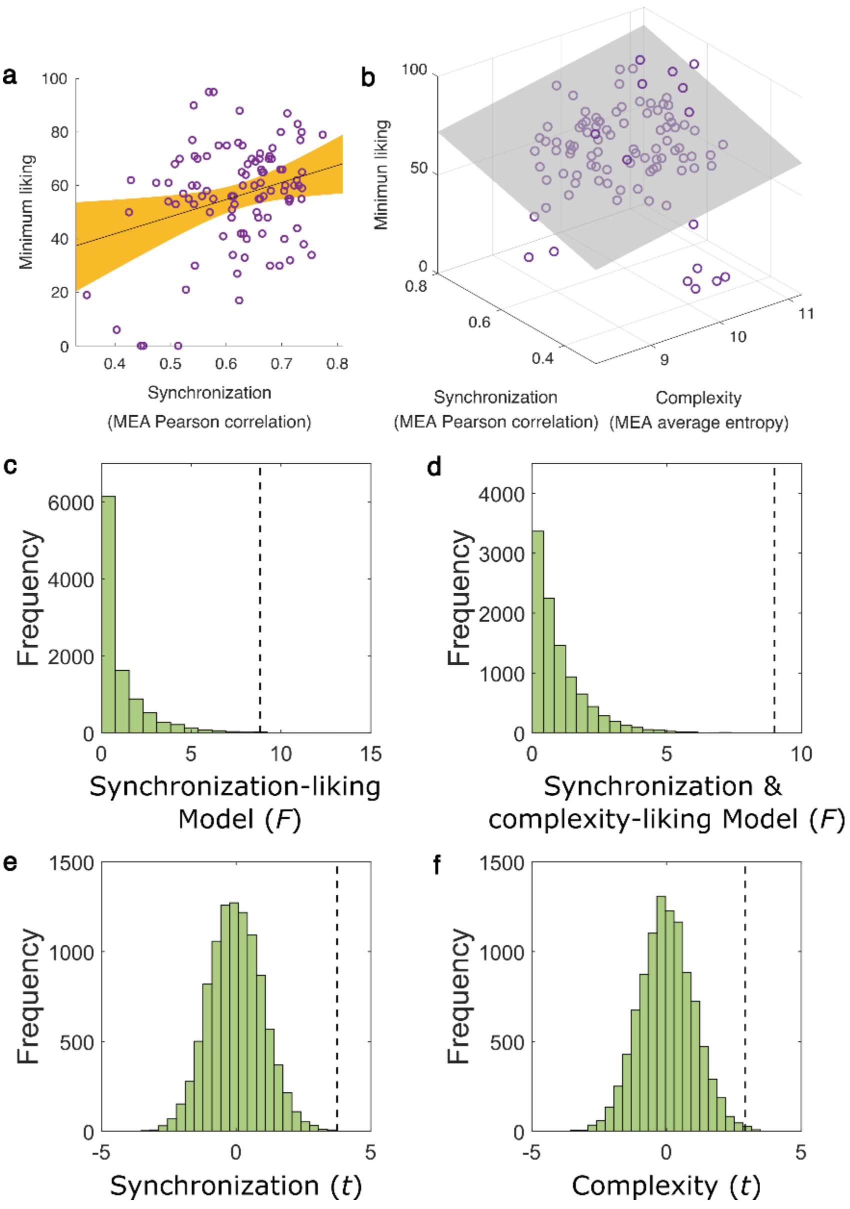

Supplementary Material Fig. 6. Predicting minimum liking by dyadic movement features. (a) Synchronization level of the z-scored MEA signals significantly predict the minimum liking within each dyad, $F(1, 98) = 8.82$, $P = .004$, permuted $P = .003$, $R^2 = .083$, $BF_{10} = 9.641$. Each circle represents a dyad, the black line is the linear regression line, and the orange area marks the confidence interval around the slope of a regression line. (b) Multiple linear regression model including both synchronization and complexity significantly improved the model predictions, this model's predicted 15.6% of the variance in the minimum liking within each dyad, $F(2, 97) = 8.995$, $P < .001$, $BF_{10} = 98.548$. Each circle represents a dyad in the 3D space and the grey plane marks the regression surface that was fitted by the model. (c) Results of permutation tests for predicting the minimum liking by synchronization and for (d) predicting the minimum liking by synchronization and complexity. Histograms depict the distribution of *F* statistics across 10,000 permutations of the data set, in which the liking ratings of each dyad were randomly shuffled. Black dashed lines depict the actual *F* statistic of each model (i.e., based on the non-permuted data). Results of these permutation tests indicate that synchronization significantly predicted minimum liking ($P = .003$), and that synchronization and complexity also predict minimum liking above chance ($P = < .001$). (e) Results of permutation tests for *t* statistic of synchronization *β* and (f) for *t* statistic of complexity *β* when predicting the minimum liking by synchronization and complexity. The histogram depicts the distribution of *t* statistics across 10,000 permutations of the data set, in which the liking ratings of each dyad were randomly shuffled. Black dashed lines depict the actual *t* statistic of each model (i.e., based on the non-permuted data). Results of these permutation tests indicate that synchronization *β* and complexity *β* are above chance, ($P < .001$ and $P = .003$ respectively).

Taking into account the small differences within each dyad (as demonstrated by the relative differences analysis), and following the high similarity between the results of the models

predicting average liking, maximum liking and minimum liking, we chose to focus on the average liking as the dyadic measure for liking.

Synchronization according to the maximal cross-correlation and Complexity Play a Role in Liking. Exploring the time domain, synchronization as measured by the maximal cross-correlation showed that in 43.2% out of the total game duration the high-liking group moved in a more synchronized manner than the low-liking group, which is significantly above chance level, binomial $P < .001$. The high-liking group moved in a significantly more synchronized manner than the low-liking group for 4.96% of the game duration. These results are denoted at the bottom of Supplementary Material Fig. 7 by the grey and black marks, respectively. In order to show by how much the probability of h_1 (the high-liking group is more synchronized than the low-liking group) is expected to be true compared to h_0, we performed Bayesian analysis. The dashed line denotes the Bayes factor (BF_{10}), which shows substantial Bayesian evidence in 2.5% of the mirror game duration.

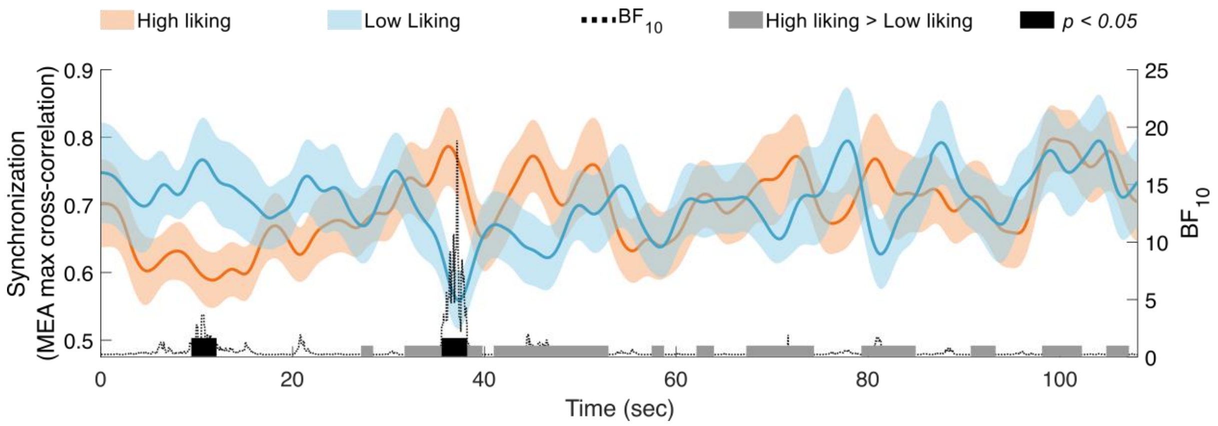

Supplementary Material Fig. 7. Maximal cross-correlation along the interaction segments for high and low-liking. Synchronization was higher in the high-liking group than the low-liking group for 43.2% of the game duration, Binomial sign test $P < .001$. The orange and blue lines denote the average value of the high and low-liking groups respectively, along the mirror game time-points. The shaded orange and blue marks denote the *SE*s of the high and low-liking groups, respectively. The gray marks at the bottom of each panel denote time-points in which the high-liking group had a higher value than the low-liking group. The black marks denote time-points in which there was a significant difference between the liking groups. The dashed lines depict two-tailed Bayes factors (BF_{10}).

Analysis of the role of Synchronization and complexity in liking using stepwise multiple linear regression. We further conducted a stepwise multiple linear regression to examine the optimal contribution of the different movement features (synchronization as measured by Pearson correlation coefficients, synchronization as measured by the maximal cross-correlation, complexity as measured by the average entropy and novelty as was measured by K-S distance) in predicting liking. The final model included the same two predictors as in the original model: synchronization as measured by Pearson correlation coefficients and average entropy. As reported before, this model predicted 14.9% of the variance in liking, R^2 = .149, F(2, 97) = 8.49, P < .001, BF_{10} = 66.541, with a positive correlation between the level of synchronization and liking, β = .35, t(97) = 3.63, P < .001, and the average entropy and liking, β = .28, t(97) = 2.84, P = .005.

Supplementary Material Table 1. Summary of the results of the multiple regression models.

Model	R^2	*F-Change* (df)	P-value	*BF*	Variable	*β*	*t* (df)	P-value	*BF*
Synchronization	.08	8.28 (1, 98)		7.67		.28	2.88 (1, 98)	.005	7.67
Synchronization + Complexity	.15	8.49 (2, 97)	< 0.001	66.54	Synchronization	.35	3.63 (2, 97)	< .001	47.97
					Complexity	.28	2.84 (2, 97)	.005	13.86
Synchronization + Complexity + Novelty	.15	.56 (1, 96)	0.456	24.51	Synchronization	.37	3.7 (1, 96)	< .001	34.62
					Complexity	.25	2.42 (1, 96)	.002	8.17
					Novelty	.08	.75 (1, 96)	0.456	1.03

Segmentation into Movements Algorithm.

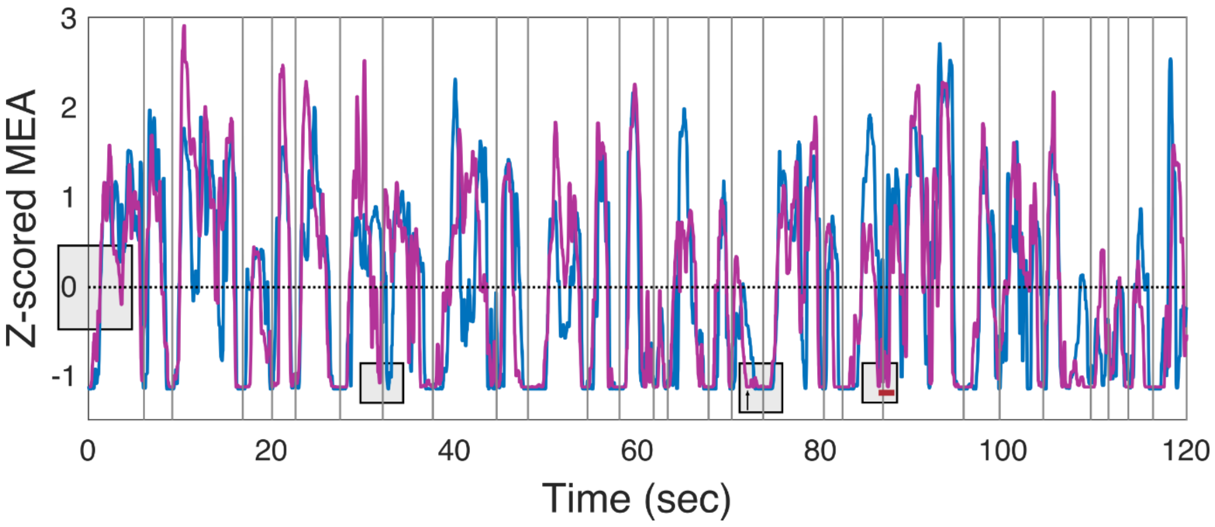

Supplementary Material Fig. 8. Example for the segmentation conditions. The z-scored MEA signals of two representative participants while playing with each other. The vertical lines denote the separation between the movement segments. This figure is identical to Fig. 1b, with added squares demonstrating the segmentation conditions. The *first square* from the left shows a case where the minimum in one signal is smaller than zero (marked by the black dashed line) and the minimum of the other signal is higher than zero, violating the condition that the value in each minimum point should be smaller than zero and thus this was not defined as a minimum point according to our conditions. The *second square* from the left shows that the time difference between the marked blue minimum and the marked magenta minimum to its left is smaller than the time difference between the marked blue minimum and the next magenta minimum to its right. In addition, the time difference between the marked magenta minimum and the marked blue minimum to its right is smaller than the time difference between the marked magenta minimum and the blue minimum to its left. Accordingly, the two marked minimum points form a shared minimum point. The *third square* from the left shows that since the signal's amplitude must change by at least 0.1 SDs between two consecutive minimum points, here we count one minimum for each player. The *fourth square* from the left demonstrates a violation of the condition that a minimum point must be in a distance of at least the length of the optimal time-window (illustrated by the red line) from the previous minimum point in the signal.

```
%%%%%%%%%%%%%%%%%%%%%%%%%%%%%%%%%%%%%%%%%%%%%%%%%
%% Segmentation_into_Movements_Algorithm - MATLAB
%%%%%%%%%%%%%%%%%%%%%%%%%%%%%%%%%%%%%%%%%%%%%%%%%

%% Note that the findpeaks() function is a part of the Signal
Processing Toolbox. Some versions of it can be found by searching
the web for "findpeaks.m"

close all
%% Set of flags that can be turned off/on
flagSmooth =1; % Smooth signals using a moving average filter.
Change to "0" to avoid filtering
flagPlotStops = 1; % Change to "0" to save time by not plotting the
signals before calculating the mutual stops denoting the upcoming
initiation of a movements

%% Collect subjects
% Add a loop in order to collect all subjects from the working
folder.
% Currently this function collects and runs on only one dyad data
file (.csv table) at time
cd '' % Set working directory within the '' to collect data files
from
directory = pwd; % Assign directory content onto a new variable
% Data of the analysed dyad will be assigned to the "s1s2" variable.
Data of the 1st participant from the dyad is located on the 2nd
column, and of the 2nd participant on the 3rd column of the matrix
% Column depicts data
s1s2 = readtable(ls([directory,'\*.csv'])); % Load dyad's data
(.csv) file, and assign it to a new variable
s1s2 = [table2array(s1s2(:,2)), table2array(s1s2(:,3))]; % convert
data table to numerical and only keep the relevant columns
(excluding indexes)

%% Main function
CreateMovementWin(s1s2, flagSmooth, flagPlotStops)

function CreateMovementWin(s1s2, flagSmooth, flagPlotSmooth)

%% Standardize and smoothing
[s1, s2] = ZscoreSBJ(s1s2); % %% Standardize participants signals
according to Z-Scores
[s1smo, s2smo, w1, w2] = smoothSBJ(s1, s2, flagSmooth); % smooth
using moving average & find moving average time window

%% Search and plot individual stops
[stop1, stop2, ind1, ind2, onlyStopInd1, onlyStopInd2] =
findLocalMin(s1smo, s2smo, w1, w2); % search for individual stops
PlotSmooth(stop1, stop2, s1smo, s2smo, ind1, ind2, flagPlotSmooth) %
plot individual stops

%% Bound the total number of stopping points according to
participants' minimum number of stopping points
[AstopInd, BstopInd, minStopSbj, maxStopNum] =
findStopNum(onlyStopInd1, onlyStopInd2);
```

```
%% Calc and plot mutual stops
[idx1, idx2] = calcMutualStops(AstopInd,BstopInd, maxStopNum); %
Look mutual stops
[idx1, idx2] = testIdx1Idx2Assignment(idx1, idx2, minStopSbj); %
Check values and whether "idx1" and "idx2" are properly assigned for
future procedures - If "s1" was assigned to "BstopInd" and to "idx2"
then change it again to be "idx1".
[ind1, ind2, stop1, stop2] = assignToLongVectors(s1smo, s2smo, idx1,
idx2); % Handle data: assign the stop indexes (idx1, idx2) to
matching NaN's vectors in the correct length (ind1, ind2) and also
assign the MEA stop values to such vectors.
PlotMutualStops(stop1, stop2, s1smo, s2smo, ind1, ind2,
flagPlotSmooth); % Plot Combined Stops

%% Plot according to the middle between both sbj stops
calcMovementsAndPlot(ind1, ind2, s1smo, s2smo)

end

function [s1, s2] = ZscoreSBJ(s1s2)% Standardize data

s1= zscore(s1s2(:,1), [], 1); s2= zscore(s1s2(:,2), [], 1);

end

function [s1smo, s2smo, w1, w2] = smoothSBJ(s1, s2, flagSmooth ) %
Smooth data

if flagSmooth
    % Smooth data and return the moving average individual windows
    [s1smo,w1] = smoothdata(s1);    [s2smo,w2] = smoothdata(s2);
end

end

function [stop1, stop2, ind1, ind2, onlyStopInd1, onlyStopInd2] =
findLocalMin(s1smo, s2smo, w1,w2) % Locate stopping points (local
minimus) within window calculated earlier according to the moving
average

% Multiple by -1 to make the local minimums to maximums. This
enables using the findpeaks() function
s1smo = -s1smo; s2smo = -s2smo;

% There is some redundancy here and in the following parts of this
function as each operation is performed on each participant in the
different code line
%SBJ1 - stopping points
[stop1, ind1] = findpeaks(s1smo,'MinPeakDistance', w1,
'MinPeakProminence',0.4);
%SBJ2 - stopping points
[stop2, ind2] = findpeaks(s2smo,'MinPeakDistance', w2,
'MinPeakProminence',0.4);

% Remove all positive stops - They are now negative

%SBJ1
```

```
ind1 = ind1(stop1>0);
stop1 = stop1(stop1> 0);

%SBJ2
ind2 = ind2(stop2>0);
stop2 = stop2(stop2> 0);

% Preallocate NaN vector for the data
temp1 = NaN(size(s1smo,1),1);      temp2 = NaN(size(s2smo,1),1);
% Assign the stops to the NaN vector
temp1(ind1) = stop1;    temp2(ind2) = stop2;
stop1 = temp1;  stop2 = temp2;
% Remove peaks, since the signal was transformed from positive to
negative all the peak values are now negative
stop1(stop1<0) = NaN;   stop2(stop2<0) = NaN;
% Inverse positivity again
stop1 = -stop1; stop2 = -stop2;
%look for the stos indexes they are not NaN's
r1 = find(~isnan(stop1)); r2=find(~isnan(stop2));
temp1 = NaN(size(s1smo,1),1);      temp2 = NaN(size(s2smo,1),1);
% Assign stops  indexes to the NaN vector
temp1(r1) = r1;    temp2(r2) = r2;
ind1 = temp1; ind2 = temp2;
onlyStopInd1 = ind1(find(~isnan(ind1)));
onlyStopInd2 = ind2(find(~isnan(ind2)));

end

function PlotSmooth(stop1, stop2, s1smo, s2smo, ind1, ind2,
flagPlotSmooth) % plot individual stops

if flagPlotSmooth
    figure();
    %% Plot dyad
    subplot(2,6,1:6);
    title('Valleys- dyad- name');
    dyadPlotNew = plot(0:size(s1smo,1)-1,s1smo,     0:size(s2smo,1)-
1,s2smo);
    set(dyadPlotNew, {'color'}, {[0.4940, 0.1840, 0.5560]; [0.8500,
0.3250, 0.0980]});
    hold on
    plot(ind1,stop1,'o','MarkerSize',8, 'MarkerEdgeColor',[ 0
0.4470    0.7410]);
    hold on
    plot(ind2,stop2,'o','MarkerSize',8,'MarkerEdgeColor',[ 0
0.4470    0.7410]);
    ylim([-2,4]);
    xlim([0, size(s1smo,1)]);
    grid on;
    title('sr1W755_W396 - (1) individualStops')
end

end

function PlotMutualStops(stop1, stop2, s1smo, s2smo, ind1, ind2,
flagPlotMutual)
```

```
    if flagPlotMutual
    %     figure();
        %% Plot dyad
        subplot(2,6,7:12);
        dyadPlotNew = plot(0:size(s1smo,1)-1,s1smo,
0:size(s2smo,1)-1,s2smo);
        set(dyadPlotNew, {'color'}, {[0.4940, 0.1840, 0.5560];
[0.8500, 0.3250, 0.0980]});
        hold on
        plot(ind1,stop1,'o','MarkerSize',8, 'MarkerEdgeColor',[ 0
0.4470    0.7410]);
        hold on
        plot(ind2,stop2,'o','MarkerSize',8,'MarkerEdgeColor',[ 0
0.4470    0.7410]);
        ylim([-2,4]);
        xlim([0, size(s1smo,1)]);
        grid on;
        title('(1) combinedStops')
    end
end

function [AstopInd, BstopInd, minStopSbj, maxStopNum] =
findStopNum(stopInd1, stopInd2)
%%  find the sbj with less stops (minStopSbj - if minStopSbj=1 then
its s1 if minStopSbj=2 then its s2), it imposes the maximum number
of stops (maxStopNum)
    [maxStopNum,minStopSbj] = min([length(stopInd1),
length(stopInd2)]);

    if minStopSbj == 1
        AstopInd = stopInd1;
        BstopInd = stopInd2;
    elseif minStopSbj ==2
        BstopInd = stopInd1;
        AstopInd = stopInd2;
    end
end

%% Function that looks for the mutual stops
function  [idx1, idx2]  = calcMutualStops(AstopInd,BstopInd,
maxStopNum)
    % Trasnpose participants vectors
    AstopInd = AstopInd';
    BstopInd = BstopInd';

    [X,Y] = meshgrid(AstopInd,BstopInd);      % Create a meshgrid
matrix, to compare stopping indexes within the each participant
    res = (abs(X-Y)); % Calc stopping indexes differences
    [~, idx2] = min(res); % Find Smallest differences - this will be
used a the mutual stopping points that will be later averaged
    idx1 = 1:length(AstopInd);
    n = 1;

    while n<length(idx2)  % Move over the stopping points and locate
according to the algorithm detailed in the methods section in the
manuscript in the "Segmentation into Movements." subsection
```

```
        if idx2(n) == idx2(n+1)
            currDiff = abs(AstopInd(idx1(n))-BstopInd(idx2(n)));
            nextDiff = abs(AstopInd(idx1(n+1))-BstopInd(idx2(n+1))
);
            if currDiff < nextDiff
                idx2(n+1) = NaN; idx1(n+1) = NaN;
            elseif currDiff > nextDiff
                if length(idx2) > maxStopNum
                   idx2(n) = NaN; idx1(n) = NaN;
                else
                   idx2(n) = NaN; idx1(n) = NaN;
                end
            elseif currDiff == nextDiff
                if idx1(n+1) > idx2(n+1)
                    res(n, n+1) = NaN;
                    [~,newMinIdx] = min(res(:,n+1));
                    idx2(n+1) = newMinIdx;
                elseif idx2(n) > idx1(n)
                    res(n+1, n+1) = NaN;
                    [~,newMinIdx] = min(res(:,n+1));
                    idx2(n+1) = newMinIdx;
                end
            end
        end
        n = n+1;
    end

    idx1 = idx1';
    idx2 = idx2';
    idx1 = AstopInd(idx1(~isnan(idx1)));
    idx2 = BstopInd(idx2(~isnan(idx2)));
    idx1 = idx1'; idx2 = idx2';

end

function [idx1, idx2] = testIdx1Idx2Assignment(idx1, idx2,
minStopSbj) % Check index assignment

    if minStopSbj==2
       temp = idx2;
       idx2 =  idx1;
       idx1 = temp;
    end
end

function [ind1, ind2, stop1, stop2] = assignToLongVectors(s1smo,
s2smo, idx1, idx2) % Create a vector that includes Nan's so it will
be easier to plot it later

    stop1 = NaN(length(s1smo), 1); stop2 = NaN(length(s2smo), 1);
    ind1 = NaN(length(s1smo), 1); ind2 = NaN(length(s2smo), 1);
    ind1(idx1) = idx1; ind2(idx2) = idx2;
    stop1(idx1) = s1smo(idx1); stop2(idx2) = s2smo(idx2);

end
```

```
function calcMovementsAndPlot(ind1, ind2, s1smo, s2smo) %% Find
final movement windows Plot according to the middle between both
participants stopping points
    ind1 = ind1(~isnan(ind1)); ind2 = ind2(~isnan(ind2));
    midInd = ceil((ind1+ind2)/2);
    figure
    title('Movements- initiate between both sbj''s stops');
    hold on
    dyadPlotNew = plot(0:size(s1smo,1)-1,s1smo,     0:size(s2smo,1)-
1,s2smo);
    set(dyadPlotNew, {'color'}, {[0.4940, 0.1840, 0.5560]; [0.8500,
0.3250, 0.0980]});
    for recIDX = 1:length(midInd)-1
        rectangle('Position',[midInd(recIDX), -2, (midInd(recIDX+1)-
1)-(midInd(recIDX)) , 6], 'EdgeColor', [0, 0.0470+(recIDX*.015),
0.7410])
    end
    ylim([-2,4]);
    xlim([0, size(s1smo,1)]);
    grid on;
end
```
